# Flexible semiparametric joint modeling: an application to estimate individual lung function decline and risk of pulmonary exacerbations in cystic fibrosis

**DOI:** 10.1186/s12982-017-0067-1

**Published:** 2017-11-14

**Authors:** Dan Li, Ruth Keogh, John P. Clancy, Rhonda D. Szczesniak

**Affiliations:** 1 Alzheimer’s Therapeutic Research Institute, Keck School of Medicine, University of Southern California, 9860 Mesa Rim Rd, San Diego, CA 92121 USA; 2 Department of Medical Statistics, London School of Hygiene and Tropical Medicine, Keppel Street, London, WC1E 7HT UK; 3 Division of Pulmonary Medicine, Cincinnati Children’s Hospital Medical Center, MLC 2021, 3333 Burnet Ave, Cincinnati, OH 45229 USA; 4 Division of Biostatistics and Epidemiology, Cincinnati Children’s Hospital Medical Center, MLC 5041, 3333 Burnet Ave, Cincinnati, OH 45229 USA

**Keywords:** Bayesian analysis, Cystic fibrosis, Functional data analysis, Longitudinal studies, Mixed model analysis, Pulmonary decline, Pulmonary function, Joint modeling, Registry analyses, Spline regression

## Abstract

**Background:**

Epidemiologic surveillance of lung function is key to clinical care of individuals with cystic fibrosis, but lung function decline is nonlinear and often impacted by acute respiratory events known as pulmonary exacerbations. Statistical models are needed to simultaneously estimate lung function decline while providing risk estimates for the onset of pulmonary exacerbations, in order to identify relevant predictors of declining lung function and understand how these associations could be used to predict the onset of pulmonary exacerbations.

**Methods:**

Using longitudinal lung function (FEV_1_) measurements and time-to-event data on pulmonary exacerbations from individuals in the United States Cystic Fibrosis Registry, we implemented a flexible semiparametric joint model consisting of a mixed-effects submodel with regression splines to fit repeated FEV_1_ measurements and a time-to-event submodel for possibly censored data on pulmonary exacerbations. We contrasted this approach with methods currently used in epidemiological studies and highlight clinical implications.

**Results:**

The semiparametric joint model had the best fit of all models examined based on deviance information criterion. Higher starting FEV_1_ implied more rapid lung function decline in both separate and joint models; however, individualized risk estimates for pulmonary exacerbation differed depending upon model type. Based on shared parameter estimates from the joint model, which accounts for the nonlinear FEV_1_ trajectory, patients with more positive rates of change were less likely to experience a pulmonary exacerbation (HR per one standard deviation increase in FEV_1_ rate of change = 0.566, 95% CI 0.516–0.619), and having higher absolute FEV_1_ also corresponded to lower risk of having a pulmonary exacerbation (HR per one standard deviation increase in FEV_1_ = 0.856, 95% CI 0.781–0.937). At the population level, both submodels indicated significant effects of birth cohort, socioeconomic status and respiratory infections on FEV_1_ decline, as well as significant effects of gender, socioeconomic status and birth cohort on pulmonary exacerbation risk.

**Conclusions:**

Through a flexible joint-modeling approach, we provide a means to simultaneously estimate lung function trajectories and the risk of pulmonary exacerbations for individual patients; we demonstrate how this approach offers additional insights into the clinical course of cystic fibrosis that were not possible using conventional approaches.

**Electronic supplementary material:**

The online version of this article (10.1186/s12982-017-0067-1) contains supplementary material, which is available to authorized users.

## Background

**Fig. 1:**
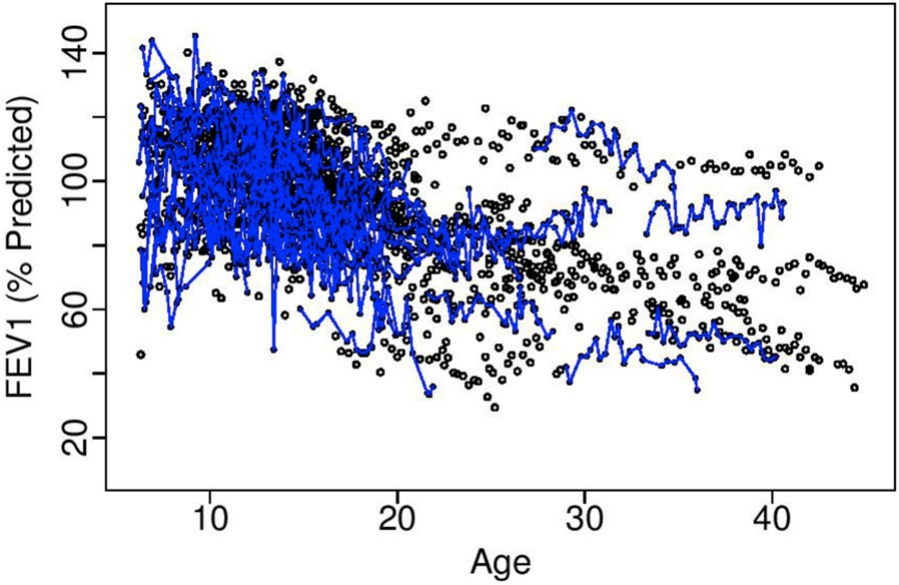
FEV1 versus age (in years) for 100 randomly selected patients with cystic fibrosis from the U.S. CFFPR, 2003–2011. Points have been connected over age for the 50 patients who were observed with pulmonary exacerbation (in blue)

Maintaining pulmonary function is essential for survival in individuals with cystic fibrosis (CF), a lung disease that currently affects nearly 70,000 individuals worldwide [1]. Lung function is measured primarily as forced expiratory volume in 1 s of percent predicted (hereafter, FEV_1_), which is the primary marker of disease severity in individuals with CF. FEV_1_ declines in a nonlinear fashion over age and exhibits substantial heterogeneity both between patients and within an individual patient over time (Fig. 1). Most individuals with CF have decreased FEV_1_ over age, but initial FEV_1_ and rate of decline vary between patients. Linear mixed modeling is an established approach to estimate age-related FEV_1_ progression. Historically, CF epidemiologic studies have not accounted for nonlinearity in the FEV_1_ trajectory; however, recent approaches have included piecewise polynomials, either in the form of regression splines or change-point models, to estimate decline in FEV_1_ [2, 3]. These approaches have shown that FEV_1_ decline is variable with maximal loss occurring in adolescence and early adulthood.

Meanwhile, the clinical course of CF is often marked by the occurrence of an acute respiratory event known as a pulmonary exacerbation, which intensifies the severity of this chronic disease. Increased pulmonary symptoms and decreased lung function and weight are common clinical indicators of this event [4]. A study of the United States Cystic Fibrosis Foundation Patient Registry showed that, after having a pulmonary exacerbation, 25% of patients’ lung function levels did not recover to baseline (pre-pulmonary exacerbation) levels [5]; this study identified lower socioeconomic status (use of Medicaid insurance), malnutrition, having respiratory infections and pathogens, and being female as risk factors. Another study identified pulmonary exacerbations as a risk factor for more rapid lung function decline [6].

Although much information has been gleaned, these and other epidemiologic studies in CF have modeled the longitudinal and time-to-event processes of lung function and pulmonary exacerbations separately. Efficiency and additional information about the disease course may be gained by modeling them simultaneously. Joint modeling of longitudinal and event processes have enjoyed a renaissance in recent years among biostatisticians and epidemiologists, largely due to software developments and modern applications demonstrating their clinical utility for the purposes of monitoring disease progression and making predictions [7]. Developments in joint models have also been motivated by data on CF disease progression, focusing on simultaneously fitting a longitudinal submodel of lung function and a survival submodel. For example, Schluchter et al. [8] developed a joint longitudinal-survival model to create prognostic indicators of CF disease severity, such as predicted age at death and other empirical Bayes estimates of parameters in the longitudinal submodel, such as slope, which could be used to estimate rate of decline in FEV_1_ adjusted for survivor bias. They combined a linear mixed effects model for longitudinal FEV_1_ with a Gaussian model for age at death and applied it to data taken from a local CF center. They later extended this model to account for left truncation, as follow-up does not necessarily begin at age zero for CF patients [9]. Such joint models can be classified as shared parameter approaches, meaning that FEV_1_ and the event of interest are assumed to be conditionally independent given a set of random effects.

In this study, we utilize the aforementioned advantages of joint modeling in the shared-parameter framework and combine them with flexible semiparametric regression splines to estimate FEV_1_ trajectories in the longitudinal submodel. In this context, joint modeling would account for informative dropout due to pulmonary exacerbation events; not accounting for these events could bias estimates of the FEV_1_ trajectories. Our objectives are to identify relevant predictors of lung function trajectories and pulmonary exacerbation events, and to understand how associations found from a joint model could be used to predict the onset of pulmonary exacerbations for individual CF patients.

## Methods

### Study design and cohort

This longitudinal cohort study consisted of clinical encounter and hospitalization data from the U.S. Cystic Fibrosis Foundation Patient Registry (CFFPR) between January 1, 2003, and December 31, 2011. This registry has been tracking CF demographic and clinical variables for more than 40 years, and has been thoroughly described elsewhere [10]. Because the majority of relevant predictors of lung function decline and pulmonary exacerbation events were consistently documented beginning in 2003, we considered data available from this year onward. Patients younger than 6 years of age were excluded due to the potential for unreliable data from pulmonary function testing. Data from those older than 45 years of age were excluded due to the possibility that these individuals have milder phenotypes that are not representative of typical CF disease progression.

The longitudinal outcome of interest, FEV_1_ % predicted (hereafter, FEV_1_), was obtained at each clinical encounter; % predicted values were calculated using Wang and Hankinson reference equations [11, 12]. The event outcome of interest, onset of first pulmonary exacerbation, was subject to censoring. A pulmonary exacerbation was considered to have occurred if documented in the CFFPR as warranting treatment with intravenous antibiotics. Analyses were restricted to patients with at least ten measurements of FEV_1_. Longitudinal data were included up to the time of the first pulmonary exacerbation on record and censored thereafter. Baseline was defined as the time point at which the first FEV_1_ measurement was available. Covariates of interest were selected from the literature on FEV_1_ [13, 14] and pulmonary exacerbation [5, 6] as separate models, and included age, gender, initial FEV_1_ measure and baseline lower socioeconomic status (lower SES, defined as having only state/federal or no insurance; recorded as 1, and 0 otherwise); a birth cohort covariate was used to adjust for potential left truncation bias; time-varying covariates included CF-related diabetes (CFRD, with or without fasting hyperglycemia), positive cultures for *methicillin*-*resistant staphylococcus aureus* (*MRSA*), *Burkholderia cepacia* (*B. cepacia*), and *Pseudomonas aeruginosa* (*Pa*).

### Joint model formulation and estimation

Each joint model consists of two linked submodels, a mixed model to fit longitudinal FEV_1_ and a Weibull model to fit pulmonary exacerbation data.

#### Longitudinal submodel for FEV_1_

Both the separate modeling approach and the joint modeling approach require specification of a model for the longitudinal measure of FEV_1_. In this subsection we describe this model before linking it to the time-to-event model in subsequent sections. Suppose there are $N$ patients in the CFFPR indexed by $i = 1,2, \ldots ,N$. Let $FEV1_{ij}$ represent lung function measured for the *i*th patient at the *j*th time, represented as $age_{ij}$ (corresponding to patient age, in years), $j = 1, \ldots ,n_{i}$. The longitudinal submodel consists of a linear mixed model with random effects:

1 $$FEV1_{ij} = f\left( {age_{ij} } \right) + m_{i} \left( {age_{ij} } \right) + W_{1i} \left( {age_{ij} } \right) + \varepsilon_{ij} ,$$

where the population-level mean response $f\left( {age_{ij} } \right)$ is modeled as a continuous function of time, which combines the time-related fixed effects terms and regression splines^15^; $m_{i} \left( {age_{ij} } \right) = {\mathbf{x}}_{1i}^{{\prime }} \left( {age_{ij} } \right)\varvec{\alpha}_{1}$ refers to the subject-level fixed effects. The vector ${\mathbf{x}}_{1i} \left( {age_{ij} } \right)$ represents static and time-varying covariates defined above, and the vector $\varvec{\alpha}_{1}$ contains their corresponding regression coefficients. The expression $W_{1i} \left( {age_{ij} } \right) = {\mathbf{z}}_{1i}^{{\prime }} \left( {age_{ij} } \right){\mathbf{U}}_{i}$ incorporates random effects that describe how subject-specific true FEV_1_ levels deviate from their expected behavior, where the vector ${\mathbf{U}}_{i}$ corresponds to subject-specific random slopes and intercepts. We used the usual Laird-Ware form^8^, $W_{1i} \left( {age_{ij} } \right) = U_{0i} + U_{1i} age_{ij}$, corresponding to ${\mathbf{z}}_{1i} \left( {age_{ij} } \right) = \left( {1,age_{ij} } \right)^{{\prime }}$; this specification allows individuals to have varying baseline FEV_1_ measurements and different rates of change in FEV_1_ over time. Lastly, the term $\varepsilon_{ij} \sim N\left( {0,\sigma_{\varepsilon }^{2} } \right)$ denotes zero-mean Gaussian measurement error.

We considered two nonlinear representations of $f\left( {age_{ij} } \right)$, which we hereafter refer to as semiparametric and cubic, and examined both in the joint modeling framework. First, we used penalized splines with a cubic truncated power basis in order to provide smooth estimates of the longitudinal course of FEV_1_ measurements. Second, we considered only global cubic polynomials to represent population-level FEV_1_ decline by excluding the basis functions in $f\left( {age_{ij} } \right)$. Details of the two formulations are available in the “Appendix 1”. Taking the first derivative with respect to age in Eq. (1) yields overall and subject-specific estimates of the rate of change in FEV_1_, and has been used previously to model FEV_1_ decline [2].

#### Time-to-event submodel for pulmonary exacerbation

Let $T_{i}$ denote the possibly censored survival time to a pulmonary exacerbation event for the *i*th patient. In a Weibull model, we assume that the survival time follows a Weibull distribution, that is $t_{i} \sim {\text{Weibull}}\left( {k, \mu_{i} \left( t \right)} \right)$, where $\mu_{i} \left( t \right) = { \exp }\left\{ {{\mathbf{x}}_{2i}^{{\prime }} \left( t \right)\varvec{\alpha}_{2} + W_{2i} } \right\}$ and $k > 0.$ The hazard at time $t$ for the *i*th patient is

2 $$h_{i} (t) = kt^{(k - 1)} \exp \left( {{\mathbf{x}}_{2i}^{{\prime }} (t)\varvec{\alpha}_{2} + W_{2i} } \right),$$

which monotonically increases with time if $k > 1$, decreases if $k < 1$, and reduces to the exponential hazard and remains constant if $k = 1$. The vectors ${\mathbf{x}}_{2i} \left( t \right)$ and $\varvec{\alpha}_{2}$ represent possibly time-dependent covariates and the corresponding regression coefficients. Covariates ${\mathbf{x}}_{2i} \left( t \right)$ need not have elements in common with ${\mathbf{x}}_{1i} \left( {age} \right)$ in the longitudinal model as shown in Eq. (1). Notice that $W_{2i}$ could have a time-dependent form [15], but we do not consider it here since this level of complexity is not required for our data. Similar to the form of $W_{1i} \left( {age} \right)$ in Eq. (1), $W_{2i} = \theta_{0} U_{0i} + \theta_{1} U_{1i}$ corresponds to patient-specific random intercepts and slopes, but has distinct regression parameter coefficients, $\theta_{0}$ and $\theta_{1}$. If none of the covariates vary over time, $W_{2i}$ reduces to 0 in the absence of random effects. The main idea of this approach is to connect the longitudinal and survival processes with a latent bivariate Gaussian process. We can add a frailty term in $W_{2}$, i.e., $W_{2i} = \theta_{0} U_{0i} + \theta_{1} U_{1i} + U_{3i}$, where $\left( {U_{0i} ,U_{1i} } \right)^{{\prime }}$ follows a bivariate Gaussian distribution, while $U_{3i} \sim N\left( {0,\sigma_{3}^{2} } \right)$, independent of $\left( {U_{0i} ,U_{1i} } \right)^{{\prime }} .$

#### Submodel links

Pulmonary exacerbation event times were associated with longitudinal FEV_1_ measurements through stochastic dependence between $W_{1i}$ and $W_{2i}$ from Eqs. (1)–(2) by assuming:

3 $$W_{1i} \left( {\text{age}} \right) = U_{0i} + U_{1i} \,{\text{age}};$$

4 $$W_{2i} \left( t \right) = W_{2i} = \theta_{0} U_{0i} + \theta_{1} U_{1i} .$$

The subject-specific random intercept and slope, depicting varying initial values and rates of FEV_1_ decline for each patient after accounting for the nonlinear FEV_1_ trajectory, are contained in the vector ${\mathbf{U}}_{i}$, which is shared between the longitudinal and time-to-event submodels. Here ${\mathbf{U}}_{i}$ is assumed to follow a bivariate normal distribution $N\left( {0,{\varvec{\Sigma}}} \right),$ where ${\varvec{\Sigma}}$ has diagonal entries $\sigma_{0}^{2}$, $\sigma_{1}^{2}$ and off-diagonal entries $\sigma_{01}$. The joint model with (4) allows both the random intercept $U_{0i}$ and slope $U_{1i}$, involved in (3), to affect the risk of the event. Thus, deviations of the patient-specific FEV_1_ trajectories from the population-level FEV_1_ curve enter the pulmonary exacerbation model in the form of random intercepts and slopes. If this type of association exists between the longitudinal FEV_1_ and pulmonary exacerbation event processes, then inference from the joint model should be less biased and more efficient, compared to modeling the processes separately [16]. Given that absolute FEV_1_ (intercept) and rate of change in FEV_1_ (slope) are on different scales, we used the estimated SDs of the random effects to express each corresponding HR as per SD change in the respective covariate. These quantities were obtained from the joint model by estimating $\exp \left\{ {\theta_{0} *\sigma_{0} } \right\}$ and $\exp \left\{ {\theta_{1} *\sigma_{1} } \right\}$, where $\theta_{0}$ and $\theta_{1}$ are the association parameters estimated ordinarily from the HRs $\exp \left\{ {\theta_{0} } \right\}$ and $\exp \left\{ {\theta_{1} } \right\}$, and $\sigma_{0}$ and $\sigma_{1}$ are the respective SDs of intercept $U_{0i}$ and slope $U_{1i}$ as defined previously.

#### MCMC sampling procedure

Conventional likelihood-based estimation of the parameters requires integration of the two submodels over the distribution of random effects. As the number of random effects in the model increase, the dimension of integration in the joint model likelihood increases; as a result, parameter estimation typically involves specialized numerical algorithms and becomes computationally burdensome [17]. Alternatively, we employed Markov-Chain Monte-Carlo (MCMC) via Gibbs sampling in WinBUGS [18] to simulate data from the respective posterior distributions under each model. The highest posterior density (HPD) and accompanying 95% credible interval (CI) were used to estimate each parameter of interest. Specification of priors and sampling procedures are detailed in the “Appendix 2”.

#### Model comparisons

We calculate the deviance information criterion (DIC) to compare the performance of the separate and joint models [19]. The DIC measure balances the fit of a model to the data with its complexity. The components of DIC for the two submodels, denoted as DIC_1_ and DIC_2_, were also provided to evaluate their relative contributions to the total DIC score. Formulas are provided in the “Appendix 3”. A smaller value of DIC indicates the preferred model.

### Research ethics

The Internal Review Board of Cincinnati Children’s Hospital Medical Center (Cincinnati, OH, USA) approved the study.

## Results

### Study cohort

There were 7672 individuals who met inclusion criteria, yielding a total of 136,051 FEV_1_ measurements. Median (IQR) follow-up was 5.8 (4.0–7.7) years; 3349 (43.7%) of the patients experienced a pulmonary exacerbation. Median age at entry was 10.8 (6.9–16.6) years; 4298 (56%) of the patients were male; 2690 (35%) had lower SES (only state/federal or no insurance). In total, 1036 (13.5%), 1176 (15.3%), 2144 (27.9%) and 3316 (43.2%) of the patients were born before 1981, between 1981 and 1988, between 1989 and 1994, and after 1994, respectively. Few patients had infection with *B. cepacia* (2.5%), but 1589 (20.7%) had *Pa* infection at baseline. Mean (SD) FEV_1_ at entry was 92.3 (19.6) % predicted.

### Longitudinal submodel for FEV_1_

Both the separate and joint modeling of longitudinal FEV_1_ indicated that decline was nonlinear over age (Table 1, Semiparametric longitudinal submodel). In both models, having higher FEV_1_ at entry corresponded to higher FEV_1_ over time; lower SES, diagnosis with CFRD and the presence of infections were associated with lower FEV_1_ over age. The younger birth cohorts appeared to have similar FEV_1_ progression, while being in an older birth cohort was associated with higher FEV_1_. Although results were consistent between the two models in Table 1, estimates from the joint model tended to be lower for some of the covariate effects. This could be attributable to incorporating the effect of pulmonary exacerbation through the shared intercept and slope terms in the joint modeling. Both models had similar variance component estimates and indicated substantial heterogeneity in the FEV_1_ response, in terms of measurement error, as well as between and within subjects.

**Table 1:** Posterior estimates for lung function decline and pulmonary exacerbation onset based on separate and joint models

Parameter	Separate model (Model I)		Joint model (Model III)	
	Posterior mean	95% HPD CI	Posterior mean	95% HPD CI
*Semiparametric longitudinal submodel for FEV*_*1*_				
Curve				
$\beta_{0}$, intercept	18.57	[16.33, 20.68]	16.89	[14.63, 20.39]
$\beta_{1}$, age	2.846	[2.545, 3.143]	3.121	[2.74, 3.382]
$\beta_{2}$, ${\text{age}}^{2}$	− 0.1295	[− 0.1393, − 0.1197]	− 0.1367	[− 0.1481, − 0.124]
$\beta_{3}$, ${\text{age}}^{3}$	− 0.0012	[− 0.0014, − 0.0009]	− 0.0016	[− 0.0017, − 0.0014]
$b_{1}$	0.0058	[0.0044, 0.0077]	0.0073	[0.0058, 0.0084]
$b_{2}$	0.0083	[0.0076, 0.0089]	0.0103	[0.0095, 0.0111]
$b_{3}$	− 0.0143	[− 0.0187, − 0.0114]	− 0.0206	[− 0.0248, − 0.0167]
$b_{4}$	− 0.0023	[− 0.0056, 0.0027]	0.0029	[− 0.001, 0.0085]
$b_{5}$	0.0041	[− 0.0015, 0.0101]	0.0015	[− 0.0033, 0.007]
$b_{6}$	− 0.0011	[− 0.012, 0.0102]	0.0008	[− 0.0095, 0.0101]
Baseline FEV1	0.6828	[0.6722, 0.6933]	0.6834	[0.6676, 0.6945]
Male	− 0.1665	[− 0.6403, 0.3137]	− 0.1650	[− 0.6054, 0.2971]
Birth cohort				
< 1981	11.37	[9.651, 12.97]	12.39	[10.89, 13.95]
1981–1988	3.766	[2.749, 4.839]	4.936	[3.995, 5.916]
1989–1994	− 0.1912	[− 0.698, 0.3584]	0.0608	[− 0.4059, 0.5908]
> 1994 (reference)				
Lower SES	− 0.6318	[− 1.075, − 0.1686]	− 0.5984	[− 1.062, − 0.1119]
CFRD	− 0.4528	[− 0.765, − 0.1465]	− 0.3695	[− 0.6731, − 0.0633]
*MRSA*	− 0.8209	[− 1.033, − 0.6121]	− 0.8114	[− 1.016, − 0.6134]
*B. cepacia*	− 0.8318	[− 1.531, − 0.1367]	− 0.7705	[− 1.441, − 0.1022]
*Pa*	− 0.5661	[− 0.6963, − 0.4314]	− 0.5673	[− 0.6971, − 0.4373]
Sources of variation				
$\sigma^{2}$ (measurement error)	59.29	[58.83, 59.76]	59.24	[58.77, 59.73]
$\sigma_{0}^{2}$ (between patients, intercept)	324.9	[307.6, 342.2]	326.8	[311, 343.8]
$\sigma_{1}^{2}$ (between patients, slope)	2.566	[2.441, 2.694]	2.602	[2.484, 2.727]
$\sigma_{01}$ (covariance, intercept and slope)	− 25.51	[− 26.82, − 24.16]	− 25.76	[− 27.11, − 24.49]
*Weibull event submodel for pulmonary exacerbation*				
$k$	2.467	[2.395, 2.537]	2.564	[2.487, 2.634]
Intercept	− 3.705	[− 3.988, − 3.416]	− 3.568	[− 3.804, − 3.296]
$\theta_{0}$ (random intercept)	–	–	− 0.0086	[− 0.0137, − 0.0036]
$\theta_{1}$ (random slope)	–	–	− 0.3532	[− 0.4104, − 0.2979]
Baseline age	− 0.0021	[− 0.0187, 0.0147]	0.0023	[− 0.0131, 0.0176]
Baseline FEV1	− 0.0158	[− 0.0178, − 0.0138]	− 0.0198	[− 0.0218, − 0.0179]
Male	− 0.2973	[− 0.3618, − 0.2314]	− 0.2940	[− 0.3606, − 0.2267]
Birth cohort				
< 1981	− 0.3785	[− 0.7538, − 0.0028]	− 0.5348	[− 0.9056, − 0.1726]
1981–1988	− 0.1805	[− 0.3947, 0.0182]	− 0.2348	[− 0.4405, − 0.0339]
1989–1994	0.1431	[0.031, 0.2544]	0.1261	[0.0178, 0.2335]
> 1994 (reference)				
Lower SES	0.1173	[0.049, 0.1872]	0.1059	[0.0382, 0.1804]

* In the Bayesian sense, a 95% CI that excludes zero indicates statistical significance at the 0.05 level. Parameters $b_{1} - b_{6}$ are regression coefficients of the cubic truncated power functions defined in “Appendix 1”

**Fig. 2:**
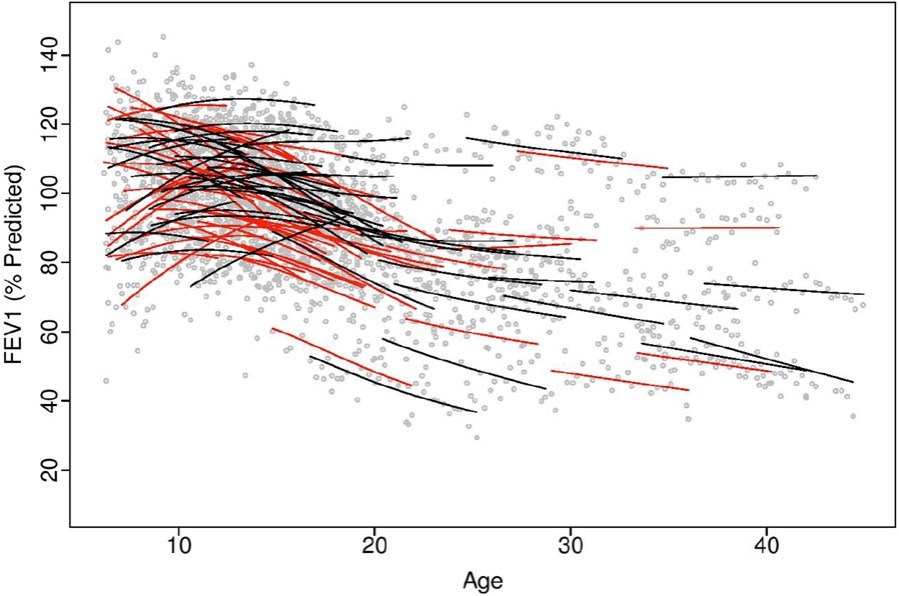
Smoothed posterior estimates of individual FEV1 for the 100 randomly selected patients presented in Fig. 1. Red lines are the smoothed estimates for individuals who were observed with pulmonary exacerbation, while black lines are the smoothed estimates for individuals who were not observed with pulmonary exacerbation

We examined the smoothed posterior estimates of individual FEV_1_ obtained from the longitudinal submodel. The estimated curves of FEV_1_ for adolescents and young adults who were observed with pulmonary exacerbation showed more rapid decline than those who had not experienced a pulmonary exacerbation (Fig. 2).

### Time-to-event submodel for pulmonary exacerbation

Both Weibull event models indicated that the hazard of pulmonary exacerbation significantly increased over age. Neither model suggested that age at entry was a significant factor (zero was an element of each 95% CI). The separate model indicates a negative association between age and risk of pulmonary exacerbation and the joint model suggests that the association was positive; however, these associations were not statistically significant. Both models imply that being male, having higher FEV_1_ at entry, and belonging to one of the earlier birth cohorts were associated with decreased risk of pulmonary exacerbation; however, being born between 1989 and 1994 corresponded to a decreased risk of pulmonary exacerbation, compared to the youngest birth cohort (those born after 1994). Lower SES was associated with an increase in the hazard of pulmonary exacerbation.

### Submodel links

Parameter estimates from the joint model indicate that lung function trajectory is associated with risk of having a pulmonary exacerbation. Formally, the intercept and slope parameters corresponding to the shared random effects in the joint model (Table 1, $\theta_{0}$ and $\theta_{1}$ estimates, respectively) imply that higher values along the FEV_1_ trajectory correspond to a decreased hazard of having a pulmonary exacerbation (HR = $\exp \left( {\hat{\theta }_{0} } \right) =$ 0.991 for every 1% predicted increase in FEV_1_, 95% CI 0.986–0.996; HR per one SD increase in FEV_1_ = 0.856, 95% CI 0.781–0.937) and positive rates of change or improvements in the FEV_1_ trajectory also correspond to decreased hazard (HR = $\exp \left( {\hat{\theta }_{1} } \right) =$ 0.702 for every increase of one percentage point (1% predicted) in the rate of change per year in FEV_1_, 95% CI 0.663–0.742; HR per one SD increase in FEV_1_ rate of change = 0.566, 95% CI 0.516–0.619). This is clinically reasonable, since a higher level of FEV_1_ represents better lung function; patients with FEV_1_ measurements that are low or with more rapid decline would be expected to have a higher hazard of pulmonary exacerbation.

### Model comparisons

**Fig. 3:**
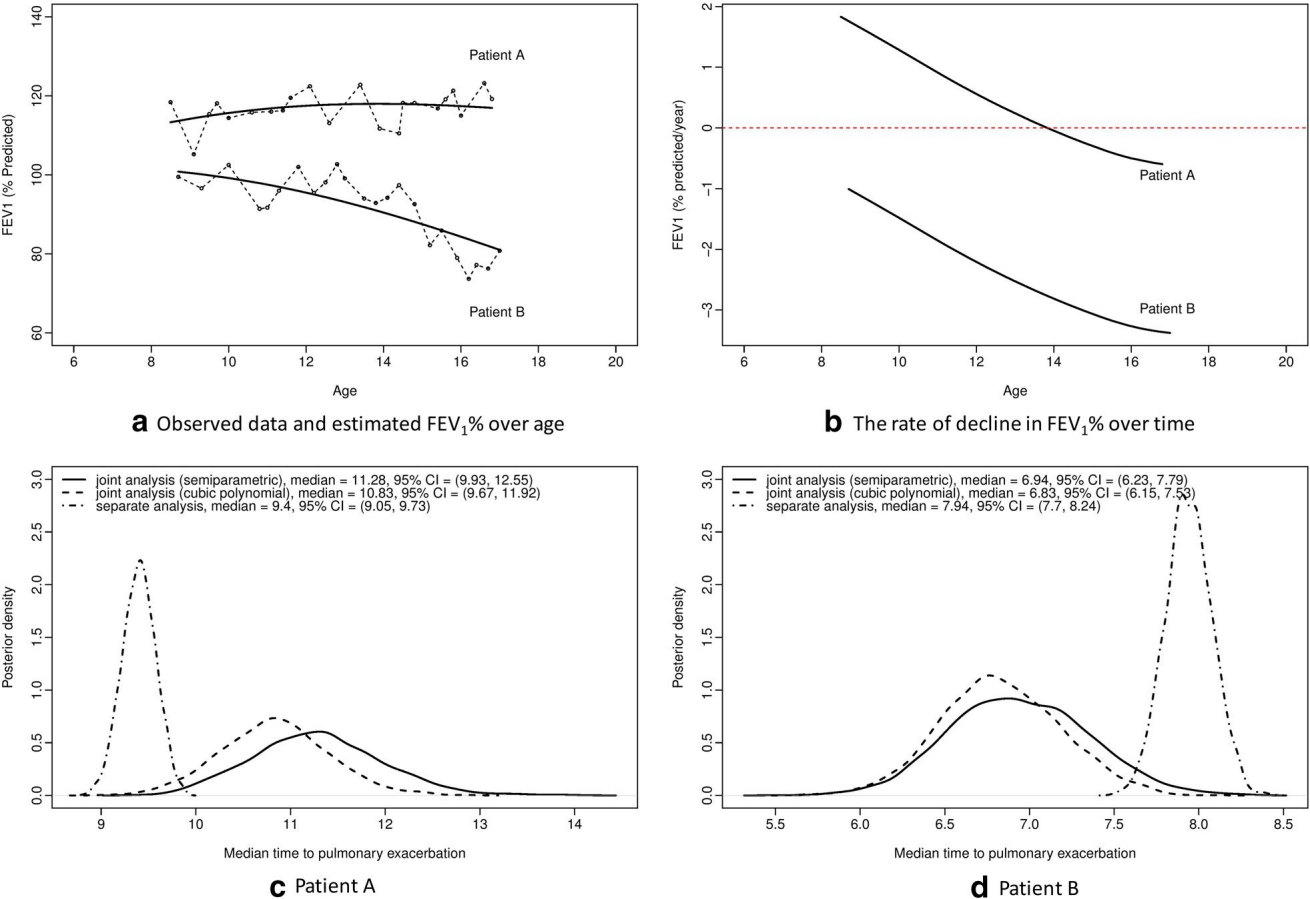
Observed data and estimated FEV1 over time (**a**), the rate of decline in FEV1 over time (**b**) and estimated posteriors of the median time to pulmonary exacerbation for two patients (**c**) and (**d**)

Obvious differences between separate and joint modeling can be graphically observed. We investigated patients with observed FEV_1_ trajectories but unknown pulmonary exacerbation event times. We selected two such patients, Patient A and Patient B and compared their estimated posterior median pulmonary exacerbation event time distributions using an established approach [20]. Although neither patient had an observed pulmonary exacerbation, they were censored at ages 16.8 and 17, respectively. Patient A’s FEV_1_ trajectory began relatively high and remained steady, but Patient B had a trajectory that started relatively low and declined over time (Fig. 3a, b). We compared the median time to pulmonary exacerbation for the two selected patients using posterior distributions from the separate pulmonary exacerbation event model and the two joint models. The predicted median time to pulmonary exacerbation for Patient A was younger under the separate model, compared to each joint model (Fig. 3c). The semiparametric joint model with cubic splines indicated that pulmonary exacerbation onset would occur at an older age than estimated for the joint model with only cubic polynomials and no splines; however, the distributions for the joint model parameters had substantial overlap. The two joint models were in closer agreement regarding time to pulmonary exacerbation for Patient B, but the estimate from the separate pulmonary exacerbation model projected a later event time (Fig. 3d). This is due to joint model’s accounting of the correlation between the longitudinal and survival data.

The semiparametric mixed effects joint model had the best fit of all models considered based on total DIC (Table 2), followed by the cubic polynomial model without splines, then the separate modeling of the longitudinal and event processes. The association between submodels through the common random intercepts and slopes in each joint model yielded a slight decrease in DIC_1_ for the longitudinal submodel and a substantial decrease in DIC_2_ for the survival submodel, compared to separate modeling.

**Table 2:** Model comparison results

Model	Submodel 1	Submodel 2	DIC_1_	DIC_2_	$\bar{D}$	*p*_D_	DIC_total_
*Separate model*							
I	Semiparametric mixed effects	Weibull	952,566	21,110.9	962,619	11,057.7	973,676
*Joint model*							
II	Cubic mixed effects	Weibull	952,915	20,712.5	962,485	11,142.6	973,628
III	Semiparametric mixed effects	Weibull	952,409	20,605.4	961,923	11,091.4	973,015

Each of DIC_1_ and DIC_2_ is the sum of the posterior mean deviance ($\bar{D}$) and the effective number of parameters ($p_{D}$); DIC_total_ obtained by summing DIC1 and DIC2 from separate or submodels (see “Appendix 4” for details). Lower values indicate better model fit

**Fig. 4:**
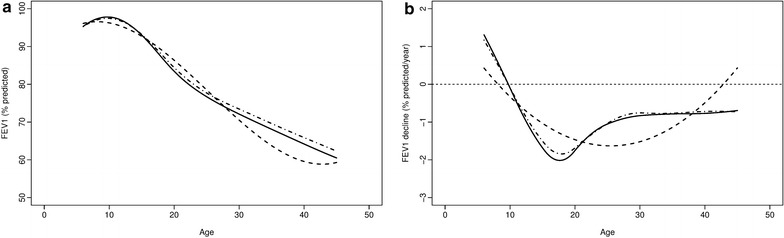
Posterior estimates obtained from joint models with semiparametric (solid line) and cubic (dashed line) submodels, and separate model with semiparametric (dash-dotted line) submodel of decline (**a**), and the rate of decline (**b**) in FEV1 over age (in years) for the overall population

In comparing the two joint models, which only differed based on inclusion of splines in the longitudinal submodel, posterior estimates of age-related FEV_1_ progression are quite similar until adolescence (Fig. 4a). Based on rates of change, the semiparametric model estimates more rapid decline into early adulthood (Fig. 4b). Although the cubic regression closely resembles the spline-based estimates, it shows an upward trend in FEV_1_ at the later range of age. Patients attained their most rapid decline at 17.6 (6.1) years; median (IQR) estimates are 17.5 (IQR 13.7–18) years. The rate of decline continues at a slower pace until patients approximately reach 30 years of age. Figure 4b indicates the cubic regression provided poorer fit and unrealistic estimates of the trend in FEV_1_ decline at the later range of age. The improvement afforded by the semiparametric model was likely due to the localized splines, which captured more variability in FEV_1_ than cubic polynomial terms alone, and in particular allowed for a more reasonable fit at later ages.

As a sensitivity analysis, we compared the joint model to a “two-stage” version of the joint model in “Appendix 5”. The two-stage model yielded similar estimates, and detected the same significant effects in the random intercept and slope as the joint model. Based on DIC, the joint model had a better fit to the data than the two-stage model, while the two-stage model was superior to the separate modeling. It demonstrates that the joint model not only allows uncertainty in the random effects to carry through to the event model, but also allows the estimation of random effects to depend on both FEV_1_ decline and the occurrence of pulmonary exacerbation.

## Discussion

In this paper, we described a flexible joint modeling technique aimed at analyzing long sequences of longitudinal and time-to-event data, and used it to simultaneously characterize the nonlinear progression of FEV_1_ and assess risk of pulmonary exacerbation events for individual CF patients. We demonstrated how this approach could be used to inform patient management regarding rapid decline in lung function and assessment of pulmonary exacerbation risk over time. On the population level, we identified clinical and demographic risk factors associated with more rapid decline in FEV_1_ and earlier onset of pulmonary exacerbation, which could be used to target subpopulations at increased risk of rapid decline or pulmonary exacerbation. Translating these individualized results into clinical care is important because, once a pulmonary exacerbation occurs, it is possible that patients will not recover to their previous FEV_1_ levels [5].

In addition to individualized estimates of pulmonary exacerbation risk that account for patient-specific longitudinal trajectories and sources of variation in FEV_1_, this study examined the effect of established risk factors for lung function decline and onset of pulmonary exacerbation. We found significant effects of birth cohort, socioeconomic status and infections with *MRSA, Pa* and *B. cepacia* on FEV_1_ decline, as well as significant effects of gender, socioeconomic status and birth cohort on pulmonary exacerbation risk. These findings corroborate what has been shown in separate modeling of FEV_1_ and pulmonary exacerbation onset [2, 5, 6]; thus, findings from joint and separate modeling of pulmonary exacerbation appear consistent on the levels of population and subpopulations stratified by risk factors. In addition, this study is the first to our knowledge that has implemented a joint modeling approach to examine the relationship between FEV_1_ decline and pulmonary exacerbation events. Through this approach, further insights are gained on an individual patient, the level at which joint and separate modeling results appear to diverge.

Our findings have a number of implications for the epidemiologic study of CF lung disease. Longitudinal modeling of FEV_1_, joint or separate, shows that there is an inverse relationship between starting FEV_1_ and rate of decline (Table 1), which corresponds to a correlation of − 0.60 between initial FEV_1_ at age 6 years (the age at which patients typically begin performing reliable pulmonary function testing) and slope estimates in our selected model. This association echoes the “ceiling effect” discovered in a previous study of pediatric CF registry data [6]. Our model setup allows us to estimate the population- and subject-specific progression of lung function decline, with the latter being important for personalized medicine. We demonstrated through a specific example that a patient with a less severe FEV_1_ trajectory is predicted to be free from a pulmonary exacerbation much longer than a patient with a more severe trajectory. It is likely that these differences among the separate and joint models regarding median pulmonary exacerbation time are attributable to the joint model’s correctly accounting for the correlation between the longitudinal and survival data. In terms of model fit, including the time-to-event submodel for pulmonary exacerbation appeared to have a greater impact than including the splines to accommodate nonlinear FEV_1_ progression, although both features improved fit (Table 2). Furthermore, having the localized differences fitted from the FEV_1_ trajectory has been shown to offer more insights into rate of decline [2, 3], particularly in examining the first derivative estimates from our study (Fig. 4). Higher-order random effects could be included to estimate more complex changes over time. Adding a frailty term to Eq. (2) did not improve the total DIC (results not shown). Similar findings have been reported regarding the fits of models with and without the frailty term, all other portions of the model being the same [17].

This study has some limitations that should be considered, which are related to survivor and delayed entry biases, reference equations for lung function and lack of a clear definition of pulmonary exacerbation. Our joint modeling approach corresponds to a missing not at random mechanism as described on pg. 90 by Rizopoulos [21]; the pulmonary exacerbation event process, which is modeled through the random effects, corresponds to the attrition mechanism in a drop-out model. There is a clear birth cohort effect on results from both separate and joint models, which has been noted in other CF registry studies [2, 14, 22] and could be reflective of advancements in care that were largely unavailable to older patients or survivor bias. Our inclusion of birth cohort in the modeling is only a partial adjustment for left truncation bias, as the correlation between age at registry entry and birth cohort will dictate the extent to which this approach combats left truncation bias. Current WinBUGS implementation is not flexible enough to specify the likelihood for each patient as being conditional on his or her entry time into the registry, which would be necessary to model the delayed entry; we refer to Crowther and colleagues’ Eq. (5) [23]. Although there are alternative R packages, such as ‘rstan’ [24], for flexible likelihood formulations to include delayed entry, estimation can be slow with a large number of patients. Faster computing algorithms would be needed to practically implement this model in large patient registry studies. FEV_1_ trajectories, which are modeled based on % predicted values over age, may differ according to the type of spirometry reference equation applied to the raw FEV_1_ data, which is expressed in liters. In terms of fitting the FEV_1_ trajectory, CFFPR data for patients aged 8–17 years taken from 2013 was analyzed in another study; Wang and Hankinson equations yielded higher median FEV_1_ values, compared to values obtained using the Global Lung Initiative equations [25]. Monitoring changes in FEV_1_, by contrast, appear to be less susceptible to this effect. There is no standard definition for what constitutes a pulmonary exacerbation; however, CFFPR studies typically infer its occurrence based on intravenous treatment with antibiotics documented in the registry [10]. This definition overlooks less severe exacerbations that impact lung function but do not warrant intravenous antibiotics. Sensitivity to this definition could be assessed with additional data from an individual CF center, which would likely have documentation on mild pulmonary exacerbation events. These events periodically occur throughout the course of CF; thus, extensions to the joint modeling approach presented here could be used to examine risk of recurrence (Additional files 1, 2).

In conclusion, we have utilized novel statistical modeling of data from a national patient registry to provide more realistic estimates of the FEV_1_ trajectory and individualized assessment of pulmonary exacerbation risk in patients with CF. Through the Bayesian approach implemented here using existing software, posterior predictive distributions of this model could be used to aid clinicians in estimating risk of pulmonary exacerbation and rate of lung function decline for individual patients. This approach could be extended to a multivariate joint model [26], in which temporal associations of the evolutions of infections and other characteristics are assessed in conjunction with lung function and exacerbations in CF patients. Furthermore, this joint modeling approach could be used to characterize lung function decline in other diseases and disorders, and to identify subgroups of individuals who may benefit from novel therapeutics being tested in clinical trials.

### Additional files

**Additional file 1.** R file for running WinBUGS from R.

**Additional file 2. ** WinBUGS file for analysis implementation.

## Appendix 1: Regression splines used in the longitudinal submodel

The function $f\left( t \right)$ is defined as a cubic truncated power basis function for the semiparametric representation:

$$f\left( t \right) = \beta_{0} + \beta_{1} t + \beta_{2} t^{2} + \beta_{3} t^{3} + \mathop \sum \limits_{l = 1}^{L} b_{l} \left( {t - \kappa_{l} } \right)_{ + }^{3} ,$$

where $\beta_{0} , \beta_{1} , \beta_{2}$ and $\beta_{3}$ are respectively the coefficients for intercept, linear, quadratic and cubic terms; the term $\sum\nolimits_{l = 1}^{L} {b_{l} \left( {t - \kappa_{l} } \right)_{ + }^{3} }$ represents basis functions for the cubic splines evaluated at time $s$, and $\left( {t - \kappa_{l} } \right)_{ + } = \hbox{max} \left( {t - \kappa_{l} , 0} \right)$. We selected the number and location of knots ($\kappa_{l} , l = 1, \ldots ,L$) for the corresponding spline basis functions using the quantile-based approach described in Ngo and Wand [27]. Please see “Appendix 2” for more discussion on knot specification. Six knots used across the [6, 45] age (in years) interval were $\varvec{\kappa}= \left( {10.78, 15.31, 19.89, 24.96, 30.49, 37.34} \right)$.

The second formulation of the longitudinal submodel defines $f\left( t \right)$ without the basis functions, which we refer to as the cubic representation:

$$f\left( t \right) = \beta_{0} + \beta_{1} t + \beta_{2} t^{2} + \beta_{3} t^{3}$$

## Appendix 2: Knot specification

The main idea of choosing knots is to choose enough knots to resolve the essential structure in the underlying regression function, but to keep the number of knots relatively low considering the computational advantages. A reasonable default is to choose the knots to ensure that there are a fixed number of unique observations (e.g., 4–5) between each knot. However, for large data sets, this may lead to an excessive number of knots, so a maximum number of knots (e.g., 20–40) is recommended. A simple default choice of $L$ that always works well is:

$$L = \hbox{max} \left\{ {5,\hbox{min} \left( {\frac{1}{4} \times {\text{number of unique}} t_{i}^{{\prime }} s, 35} \right)} \right\}.$$

A reasonable default rule for the knot location is:

$$K_{l} = \{ (l + 1)/(L + 2)\} {\text{th}}\,{\text{sample}}\,{\text{quantile}}\,{\text{of}}\,{\text{the}}\,{\text{unique's}},$$

for $l = 1, \ldots ,L$. The default choice of knots can be generated in R using the function defined as below:

## Appendix 3: Prior specification and sampling

We used a fully Bayesian version of the joint modeling approach implemented via Markov chain Monte Carlo (MCMC) method to fit the joint model. Although we focus on a particular form, the Bayesian framework allows for easier comparison among many forms of association between $W_{1}$ and $W_{2}$. The data analyses are conducted using MCMC through WinBUGS software [28]. R and WinBUGS codes implementing the proposed method are available in Additional files 1 and 2. We selected vague prior distributions in the Bayesian analysis. For each of the regression coefficients of the cubic penalized spline and the subject-level covariate effects $\varvec{\beta}, \varvec{b}$ and $\varvec{\alpha}_{1}$, we used a multivariate normal prior with mean vector $0$ and precision matrix $0.01 \cdot {\mathbf{I}}$, where ${\mathbf{I}}$ is the identity matrix; and an inverse gamma prior^19^ $IG\left( {0.01,0.01} \right)$ for the measurement error variance $\sigma_{\varepsilon }^{2}$. Similarly, we selected a vague normal prior in the survival submodel, $\varvec{\alpha}_{1} \sim N\left( {0,\varvec{ }0.01 \cdot {\mathbf{I}}} \right)$. For the random effects ${\mathbf{U}}_{i}$ common to both submodels, we took a non-informative multivariate inverse-Wishart distribution with degrees of freedom equals to 2 (the number of random effect components). Finally, for the association parameters, we set $\theta_{0} \sim N\left( {0,100} \right)$ and $\theta_{1} \sim N\left( {0,100} \right)$.

Complete conditional distributions were used to approximate the posterior distributions for the parameters of interest. MCMC diagnostics to assess convergence were performed in accordance with standard techniques [29]. Early samples were discarded based on graphical inspection of burn-in across MCMC iterations. The 95% credible intervals (CIs) were obtained by taking the 2.5th and 97.5th percentiles of the posterior samples post burn-in. For each model, we conducted the MCMC simulation for 40,000 iterations. The first 20,000 draws were discarded as a burn-in phase. The effective iterations were thinned by storing every 10th iteration in order to reduce level of correlation between successive values of the sample. The posterior mean and 95% confidence interval (CI) were calculated with the resulting 2000 values. The computing time for each model is shown in Table 3.

**Table 3:** Computing time for each model in WinBUGS

Model	Submodel 1	Submodel 2	Computing time
*Separate model*			
I	Semiparametric mixed effects	Weibull	9 h 57 min
*Joint model*			
II	Cubic mixed effects	Weibull	8 h 45 min
III	Semiparametric mixed effects	Weibull	11 h 14 min

* The data set contains 7672 individuals, and the total number of longitudinal observations (FEV_1_ measurements) is 36,051. For each model, we run 40,000 iterations, discard the first 20,000 as a warm-up phase and thin by 10, yielding a total of 2000 posterior samples

## Appendix 4: DIC formulation

Each deviance information criterion (DIC) for assessing model fit is calculated as ${\text{DIC}} = D + p_{D}$, with the posterior mean of deviance $D$ penalized by the effective number of parameters $p_{D}$ under the Bayesian framework. Since small $D$ indicates good fit while small $p_{D}$ indicate a parsimonious model, smaller values indicate better model fit, compared to larger values. We respectively write the components of DIC for the longitudinal and survival submodels as ${\text{DIC}}_{1}$ and ${\text{DIC}}_{2}$, which evaluate the contributions to the overall DIC, denoted as ${\text{DIC}}_{\text{total}}$ in Table 2. All DIC values are output directly from WinBUGS.

## Appendix 5: Comparison between two-stage model and joint model

In the joint modeling literature, various two-stage methods have been proposed. Here we compare the predictive power of a joint model with that of a simple (naïve) two-stage model. The naïve two-stage model consists of: (Stage I) longitudinal model for FEV_1_; and (Stage II) Weibull model for pulmonary exacerbation with random effects from Stage I as covariates. To be consistent with the joint model (Model III), we use the same semiparametric mixed effects modeling for FEV_1_. The posterior means of subject-specific random effects (i.e., random intercept $\tilde{U}_{0i}$ and slope $\tilde{U}_{1i}$) from Stage I are included as covariates in the Weibull model in Stage II. The parameters of the two-stage model are given the same priors as the joint model, and are estimated using the fully Bayesian-MCMC (WinBUGS) approach. The results of posterior estimates and model comparison for two-stage model and joint model (Model III) are given in Tables 4 and 5 .

**Table 4:** Posterior estimates under two-stage model and joint model for lung function decline and pulmonary exacerbation onset

Parameter	Two-stage model		Joint model (Model III)	
	Posterior mean	95% HPD CI	Posterior mean	95% HPD CI
*Semiparametric longitudinal submodel for FEV*_*1*_				
Curve				
$\beta_{0}$, intercept	18.15	[15.54, 20.95]	16.89	[14.63, 20.39]
$\beta_{1}$, age	2.86	[2.477, 3.189]	3.121	[2.74, 3.382]
$\beta_{2}$, age^2^	− 0.1306	[− 0.1418, − 0.117]	− 0.1367	[− 0.1481, − 0.124]
$\beta_{3}$, age^3^	− 0.0011	[− 0.0013, − 0.0009]	− 0.0016	[− 0.0017, − 0.0014]
$b_{1}$	0.0051	[0.0039, 0.0061]	0.0073	[0.0058, 0.0084]
$b_{2}$	0.0108	[0.0097, 0.0119]	0.0103	[0.0095, 0.0111]
$b_{3}$	− 0.0177	[− 0.0226, − 0.0133]	− 0.0206	[− 0.0248, − 0.0167]
$b_{4}$	− 0.0001	[− 0.0044, 0.0065]	0.0029	[− 0.001, 0.0085]
$b_{5}$	0.0033	[− 0.0024, 0.0083]	0.0015	[− 0.0033, 0.007]
$b_{6}$	− 0.0009	[− 0.0114, 0.0095]	0.0008	[− 0.0095, 0.0101]
Baseline FEV1	0.686	[0.6728, 0.6978]	0.6834	[0.6676, 0.6945]
Male	− 0.15	[− 0.582, 0.2736]	− 0.1650	[− 0.6054, 0.2971]
Birth cohort				
< 1981	11.43	[9.837, 13.29]	12.39	[10.89, 13.95]
1981–1988	4.0	[2.992, 4.978]	4.936	[3.995, 5.916]
1989–1994	− 0.1924	[− 0.7038, 0.3305]	0.0608	[− 0.4059, 0.5908]
> 1994 (reference)				
Lower SES	− 0.623	[− 1.091, − 0.175]	− 0.5984	[− 1.062, − 0.1119]
CFRD	− 0.4613	[− 0.7895, − 0.1506]	− 0.3695	[− 0.6731, − 0.0633]
*MRSA*	− 0.816	[− 1.026, − 0.6022]	− 0.8114	[− 1.016, − 0.6134]
*B. cepacia*	− 0.7964	[− 1.465, − 0.1291]	− 0.7705	[− 1.441, − 0.1022]
*Pa*	− 0.5643	[− 0.6953, − 0.4345]	− 0.5673	[− 0.6971, − 0.4373]
Sources of variation				
$\sigma^{2}$ (measurement error)	59.29	[58.83, 59.78]	59.24	[58.77, 59.73]
$\sigma_{0}^{2}$ (between patients, intercept)	324.1	[307.6, 341.3]	326.8	[311, 343.8]
$\sigma_{1}^{2}$ (between patients, slope)	2.564	[2.443, 2.69]	2.602	[2.484, 2.727]
$\sigma_{01}$ (covariance, intercept and slope)	− 25.46	[− 26.81, − 24.18]	− 25.76	[− 27.11, − 24.49]
*Weibull event submodel for pulmonary exacerbation*				
$k$	2.524	[2.456, 2.601]	2.564	[2.487, 2.634]
intercept	− 3.509	[− 3.802, − 3.26]	− 3.568	[− 3.804, − 3.296]
$\theta_{0}$ (random intercept)	− 0.0098	[− 0.0149, − 0.0048]	− 0.0086	[− 0.0137, − 0.0036]
$\theta_{1}$ (random slope)	− 0.3273	[− 0.3825, − 0.2728]	− 0.3532	[− 0.4104, − 0.2979]
Baseline age	0.0002	[− 0.0136, 0.0161]	0.0023	[− 0.0131, 0.0176]
Baseline FEV1	− 0.0193	[− 0.0211, − 0.0175]	− 0.0198	[− 0.0218, − 0.0179]
Male	− 0.2919	[− 0.36, − 0.2194]	− 0.2940	[− 0.3606, − 0.2267]
Birth cohort				
< 1981	− 0.5046	[− 0.8512, − 0.1807]	− 0.5348	[− 0.9056, − 0.1726]
1981–1988	− 0.2297	[− 0.4382, − 0.0415]	− 0.2348	[− 0.4405, − 0.0339]
1989–1994	0.1171	[0.0086, 0.2197]	0.1261	[0.0178, 0.2335]
>1994 (reference)				
Lower SES	0.1091	[0.0369, 0.1799]	0.1059	[0.0382, 0.1804]

**Table 5:** Model comparison results

Model	Submodel 1	Submodel 2	DIC_1_	DIC_2_	$\bar{D}$	$p_{D}$	DIC_total_
Two-stage model	Semiparametric mixed effects	Weibull	952,553	20,740.6	951,177	11,063.7	973,294
Joint model (Model III)	Semiparametric mixed effects	Weibull	952,409	20,605.4	961,923	11,091.4	973,015

The two-stage model yields similar estimates, and detects the same significant effects in the random intercept and slope as the joint model. However, DIC in Table A3 suggests that the joint model (DIC_total_ = 973,015) gives a much better fit of the data than the two-stage model (DIC_total_ = 973,294).

## Abbreviations

*B. cepacia*: *Burkholderia cepacia*
CF: Cystic fibrosis
CFFPR: Cystic fibrosis foundation patient registry
CFRD: Cystic fibrosis related diabetes
*MRSA*: *Methicillin*-*resistant staphylococcus aureus*
*Pa*: *Pseudomonas aeruginosa*
SES: Socioeconomic status

## References

[1] Farrell PM (2008). The prevalence of cystic fibrosis in the European Union. J Cyst Fibros.

[2] Szczesniak RD (2013). A semiparametric approach to estimate rapid lung function decline in cystic fibrosis. Ann Epidemiol.

[3] Moss A (2016). A comparison of change point models with application to longitudinal lung function measurements in children with cystic fibrosis. Stat Med.

[4] Ferkol T, Rosenfeld M, Milla CE (2006). Cystic fibrosis pulmonary exacerbations. J Pediatr.

[5] Sanders DB (2010). Failure to recover to baseline pulmonary function after cystic fibrosis pulmonary exacerbation. Am J Respir Crit Care Med.

[6] Konstan MW (2007). Risk factors for rate of decline in forced expiratory volume in one second in children and adolescents with cystic fibrosis. J Pediatr.

[7] Asar O (2015). Joint modelling of repeated measurement and time-to-event data: an introductory tutorial. Int J Epidemiol.

[8] Schluchter MD, Konstan MW, Davis PB (2002). Jointly modelling the relationship between survival and pulmonary function in cystic fibrosis patients. Stat Med.

[9] Piccorelli AV, Schluchter MD (2012). Jointly modeling the relationship between longitudinal and survival data subject to left truncation with applications to cystic fibrosis. Stat Med.

[10] Knapp, E.A., et al., *The Cystic Fibrosis Foundation Patient Registry. Design and Methods of a National Observational Disease Registry.* Ann Am Thorac Soc, 2016;13(7):1173–79. 10.1513/AnnalsATS.201511-781OC 27078236

[11] Wang X (1993). Pulmonary function between 6 and 18 years of age. Pediatr Pulmonol.

[12] Hankinson JL, Odencrantz JR, Fedan KB (1999). Spirometric reference values from a sample of the general US population. Am J Respir Crit Care Med.

[13] Corey M (1997). Longitudinal analysis of pulmonary function decline in patients with cystic fibrosis. J Pediatr.

[14] Taylor-Robinson D (2012). Understanding the natural progression in %FEV1 decline in patients with cystic fibrosis: a longitudinal study. Thorax.

[15] Henderson R, Diggle P, Dobson A (2000). Joint modelling of longitudinal measurements and event time data. Biostatistics.

[16] Tsiatis AA, Davidian M (2004). Joint modeling of longitudinal and time-to-event data: an overview. Stat Sin.

[17] Fitzmaurice G, Davidian M, Verbeke G, Molenberghs G, Molenberghs G, Fitzmaurice G (2009). Longitudinal data analysis. Incomplete data: introduction and overview.

[18] Lunn DJT, Best N, Spiegelhalter D (2000). WinBUGS: a Bayesian modelling framework—concepts, structure, and extensibility. Stat Comput.

[19] Spiegelhalter DJ, Best NGC, Van der Linde A (2002). Bayesian measures of model complexity and fit (with discussion). J R Stat Soc Ser B.

[20] Guo XC, Carlin BP (2004). Separate and joint modeling of longitudinal and event time data using standard computer packages. Am Stat.

[21] Rizopoulos D (2012). Joint models for longitudinal and time-to-event data: with applications in R.

[22] VanDevanter DR, Pasta DJ, Konstan MW (2014). Improvements in lung function and height among cohorts of 6-year-olds with cystic fibrosis from 1994 to 2012. J Pediatr.

[23] Crowther MJ (2016). Joint modelling of longitudinal and survival data: incorporating delayed entry and an assessment of model misspecification. Stat Med.

[24] Stan Development Team. Rstan: the R interface to Stan, version 2.16.2; 2017. http://mc-stan.org. Accessed 9 Nov 2017.

[25] Foundation CF, Cystic fibrosis foundation patient registry. In: 2013 annual report to the center directors. Cystic Fibrosis Foundation: Bethesda; 2014.

[26] Albert PS, Shih JH (2010). An approach for jointly modeling multivariate longitudinal measurements and discrete time-to-event data. Ann Appl Stat.

[27] Ngo L, Wand M (2004). Smoothing with mixed model software. J Stat Softw.

[28] Lunn DJ, Thomas A, Best N, Spiegelhalter D (2000). WinBUGS—a Bayesian modelling framework: concepts, structure, and extensibility. Stat Comput.

[29] Spiegelhalter D, Thomas A, Best N, Gilks W. WinBUGS user manual. http://www.mrc-bsu.cam.ac.uk/wp-content/uploads/manual14.pdf.

